# Adipose tissue macrophages and their role in obesity-associated insulin resistance: an overview of the complex dynamics at play

**DOI:** 10.1042/BSR20220200

**Published:** 2023-03-09

**Authors:** Suktara Guria, Anupama Hoory, Snehasis Das, Dipanjan Chattopadhyay, Sutapa Mukherjee

**Affiliations:** Endocrinology & Metabolism Laboratory, Department of Zoology, Siksha Bhavana (Institute of Science), Visva-Bharati (A Central University), Santiniketan 731235, West Bengal, India

**Keywords:** Adipose tissue, Adipose tissue macrophages, inflammation, insulin resistance, Obesity

## Abstract

Obesity, a major global health concern, is characterized by serious imbalance between energy intake and expenditure leading to excess accumulation of fat in adipose tissue (AT). A state of chronic low-grade AT inflammation is prevalent during obesity. The adipose tissue macrophages (ATM) with astounding heterogeneity and complex regulation play a decisive role in mediating obesity-induced insulin resistance. Adipose-derived macrophages were broadly classified as proinflammatory M1 and anti-inflammatory M2 subtypes but recent reports have proclaimed several novel and intermediate profiles, which are crucial in understanding the dynamics of macrophage phenotypes during development of obesity. Lipid-laden hypertrophic adipocytes release various chemotactic signals that aggravate macrophage infiltration into AT skewing toward mostly proinflammatory status. The ratio of M1-like to M2-like macrophages is increased substantially resulting in copious secretion of proinflammatory mediators such as TNFα, IL-6, IL-1β, MCP-1, fetuin-A (FetA), etc. further worsening insulin resistance. Several AT-derived factors could influence ATM content and activation. Apart from being detrimental, ATM exerts beneficial effects during obesity. Recent studies have highlighted the prime role of AT-resident macrophage subpopulations in not only effective clearance of excess fat and dying adipocytes but also in controlling vascular integrity, adipocyte secretions, and fibrosis within obese AT. The role of ATM subpopulations as friend or foe is determined by an intricate interplay of such factors arising within hyperlipidemic microenvironment of obese AT. The present review article highlights some of the key research advances in ATM function and regulation, and appreciates the complex dynamics of ATM in the pathophysiologic scenario of obesity-associated insulin resistance.

## Introduction

Since the first account by Elie Metchnikoff in 1882, macrophages, the phagocytic sentinel cells of the body, have come a long way [1]. Originally described as innate immune cells that form the first line of host defense against invading foreign pathogens, toxins, allergens, and various xenobiotics, macrophages have been found to play key roles in clearing cell debris and damaged tissue, activating and resolving sterile inflammation as well as in healing, tissue regeneration, and repair processes. These ubiquitous professional phagocytes possess an array of receptors that can respond to a variety of molecular signals arising from either pathogens or damaged tissue. Armed with a repertoire of pattern recognition receptors including Toll-like receptors (TLRs), C-type lectin receptors (CLRs), nucleotide-binding oligomerization domain (NOD)-like receptors (NLRs), retinoic acid-inducible gene I (RIG-I)-like helicase receptors (RLRs) and scavenger receptors, macrophages can sense a myriad of pathogen-associated and/or damage-associated molecular patterns (PAMPs and DAMPs, respectively), and mediate immune responses [2,3]. These highly heterogeneous cells possess diverse phenotypes and a dynamic secretory profile comprising different growth factors, cytokines and chemokines. The wide-ranging plasticity displayed by macrophages in co-ordinating responses to various cues emanating in the tissue niche has been fascinating researchers in the recent years [4]. Not only in maintaining homeostasis in normal health but the crucial role of these immune cells in chronic sterile inflammatory conditions viz. allergy [5], autoimmunity [6], cancer [7], and obesity-induced insulin resistance [8,9] are being increasingly recognized. The present study is focused on the role of macrophages in the context of obesity-induced insulin resistance and its related metabolic impairment.

## Obesity-associated insulin resistance

Obesity, a condition arising from an imbalance between energy intake and energy expenditure with the balance tipping toward the former, poses major risk for type 2 diabetes (T2D), endothelial dysfunction, and cardiovascular diseases. A complex interplay of endocrine, inflammatory, and neuronal pathways links obesity with insulin resistance [10]. The loss of sensitivity to the hormone insulin in specific target cells, primarily adipocytes, hepatocytes, and myocytes, is an inherent pathologic feature of obesity and its associated metabolic diseases such as T2D, nonalcoholic fatty liver disease, hypertension, dyslipidemia, cardiovascular diseases, and even cancer. In insulin-resistant condition, the biological action of insulin to maintain glucose and lipid homeostasis is compromised that gradually leads to the onset of hyperglycemia and hyperlipidemia with manifestations of T2D. Initially, this is compensated by overactivity of the pancreatic β cells. However, in the long-term such compensatory enhanced insulin secretion exhausts the pancreatic β-cell population, which ultimately cease to function. T2D is associated with reduced expression of insulin receptor (IR) and also defective signaling. The disruption in insulin-signaling pathway adversely affects hormone-induced metabolic responses and glucose uptake in the peripheral tissues. Adipose tissue (AT) either in excess or when lost in lipodystrophies, is intimately connected with insulin resistance and its complications [11].

Ectopic lipid metabolites and innate immune pathways have long been noted to be linked with insulin resistance [12]. Several protein kinase C (PKC) isoforms are involved in saturated fatty acid (SFA)-mediated insulin resistance; for example, IR down-regulation accompanied overexpression of PKCε [13] and insulin receptor substrate-1 (IRS-1) inactivation could arise from PKCδ-induced serine phosphorylation [14]. Besides, excess SFA could induce phosphorylation of PKCε in a kinase-independent manner through palmitoylation; pPKCε upon nuclear translocation phosphorylated an architectural transcription factor, high mobility group A1 (HMGA1) that in turn, hindered its mobility to activate IR promoter and subsequently lowered IR expression [15–17]. Exposure to SFA caused accumulation of lipid metabolites such as ceramide, which could block insulin signaling [18]. SFA not only dampened insulin-stimulated IRS-1 tyrosine phosphorylation and phosphatidylinositol 3-kinase (PI3K) activity but also enhanced IRS-1 serine phosphorylation affecting downstream signal transduction [19]. Furthermore, SFA caused GLUT4 translocation defects leading to deficiency of cellular glucose uptake [20].

Another potent inhibitor of IR tyrosine kinase phosphorylation and downstream insulin signal transduction is the hepatic secretory glycoprotein, fetuin-A (FetA), also known as alpha-2-Heremans-Schmid glycoprotein (AHSG) [21,22]. The synthesis of FetA from liver cells could be significantly augmented by SFA in nuclear factor-κB (NF-κB)-dependent manner [23]. Obese diabetic patients and high-fat diet-fed animals displayed consistently elevated levels of circulating FetA [24,25]. Polymorphism in the gene-encoding human FetA was closely linked to impaired insulin action in adipocytes [26]. Besides liver, AT [27,28] and pancreatic β cells [29] were also reported as potential sources of FetA in hyperlipidemic condition generated by excess SFA. Research over the past decade has well highlighted the close link between FetA, adiposity, innate immunity, and inflammation. During obesity, a state of chronic low-grade tissue inflammation is prevalent in AT and several other tissues including liver, skeletal muscle, pancreatic islets, and brain [30–34]. In particular, the potential role of AT inflammation in the context of obesity-associated insulin resistance and its multiple complications has been extensively studied, which will be discussed in the following sections.

## AT inflammation in obesity

AT is broadly classified into white adipose tissue (WAT) and brown adipose tissue (BAT). WAT comprises 5–50% of the total body weight and is localized in subcutaneous, visceral, epicardial, and perivascular depots; the percentage of BAT is inversely proportional with age [35,36]. An intermediate type, namely beige AT, was also found to improve insulin sensitivity by increasing energy drainage as well as absorption of glucose and lipids from blood [37]. Besides maintaining energy homeostasis, AT is a vital endocrine organ and in particular, the endocrine signals emanating from WAT can influence various other organs and control almost whole-body metabolism [38]. In obesity, AT becomes dysfunctional and mounting evidences have indicated that mostly visceral fat contributes to systemic inflammation, insulin resistance, and metabolic syndrome [35].

Pioneering studies in early 1990s provided a direct mechanistic link between AT and inflammation. High-tumor necrosis factor α (TNFα) expression was reported in AT obtained from different genetically altered diabetic and obese rodent models, and also in human samples [39,40], and neutralization of TNFα in obese rodents dramatically improved insulin sensitivity [39]. Furthermore, TNFα neutralization markedly restored insulin-stimulated autophosphorylation activity of IR as well as phosphorylation of IRS-1. Later, the role of TNFα in eliciting apoptosis in brown adipocytes and impairment of its function was reported [41]. TLR4 expressed in both adipocytes and macrophages is a key mediator of fatty acid-induced insulin resistance [42]. Originally known in *Drosophila* for establishing dorso-ventral pattern in the developing embryo [43], TLRs were later identified as important pattern recognition receptors-mediating innate immunity. Different isoforms of TLRs responding to specific microbial ligands are expressed by immune cells such as macrophages. Besides, TLR4 receptors can respond to certain endogenous ligands. Interestingly, FetA was demonstrated to act as an adaptor protein binding to SFA and then presenting it to TLR4 on the membrane of adipocytes; this ternary complex-activated NF-κB subsequently releasing an array of proinflammatory cytokines, TNFα and interleukins, IL-6 and IL-1β [44]. A state of low-grade chronic inflammation persists in obesity-mediated insulin resistance the source of which is the lipid-laden hypertrophic adipocytes and also the macrophages residing within inflamed AT.

## Adipose tissue macrophage dynamics in obesity

**Figure 1:**
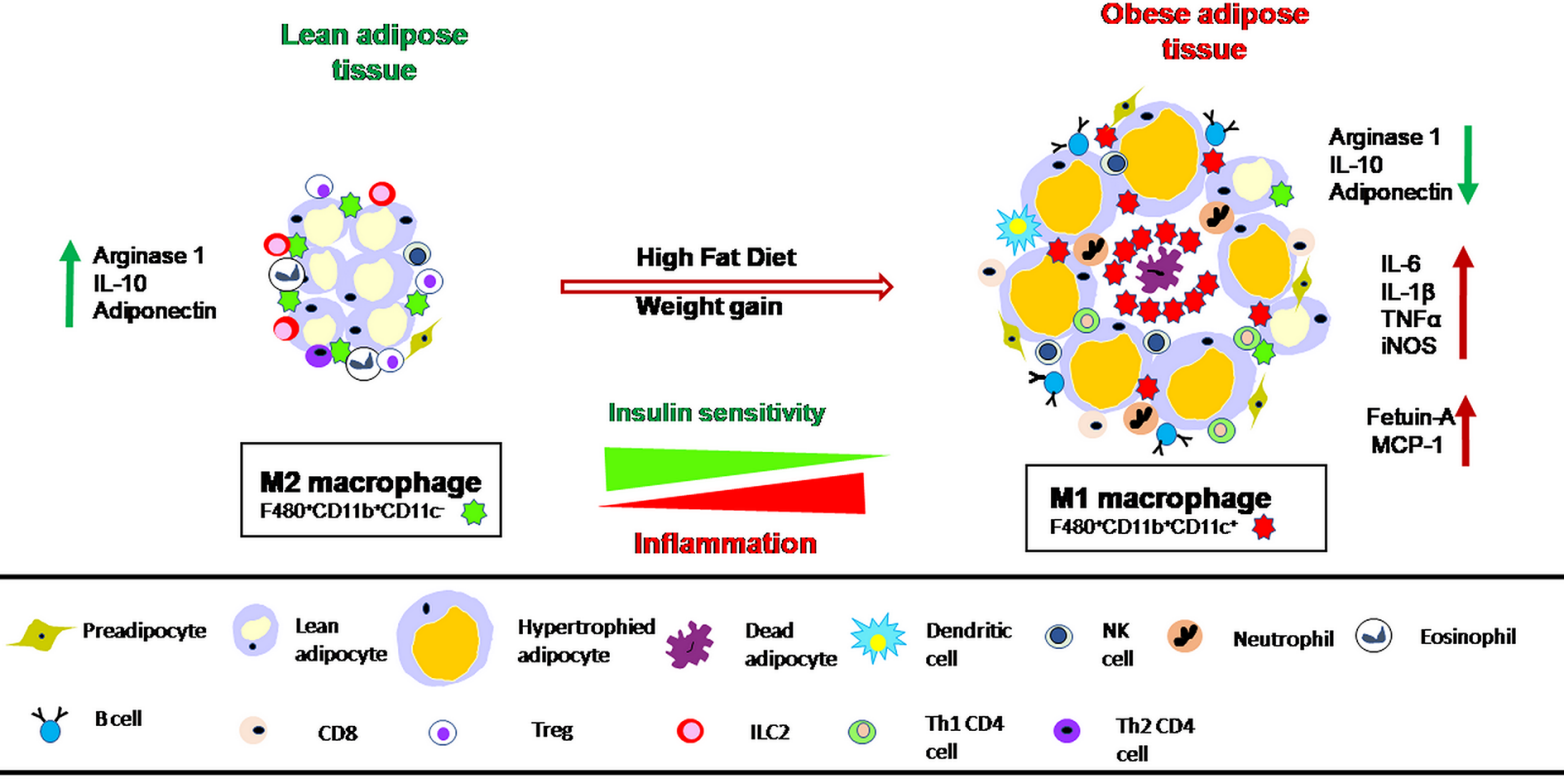
Profile of ATM phenotypes in lean and obese states Due to high-fat diet feeding, energy equilibrium shifts toward energy storage in AT, leading to enhanced adiposity and inflammation, and subsequent loss of insulin sensitivity. The adipokine secretory profile is altered that attains majorly M1-like phenotype with upregulated expression of proinflammatory cytokines in obese condition accompanied with prominent crown-like structure (CLS) formation surrounding dead adipocytes. On the other hand, M2-like subtype is predominantly found in lean AT characterized by enhanced secretion of mostly anti-inflammatory cytokines.

AT is heterogeneous in nature. Besides adipocytes, the chief fat-storing cells, it contains several other cell types including preadipocytes, endothelial cells, and various types of immune cells [45]. Macrophages are the most abundant type of leukocytes in AT. The content and characteristics of adipose tissue macrophages (ATM) undergo dramatic changes in obese condition (Figure 1). The first insight into the role of macrophages in obesity came from extensive research carried out by two independent groups in 2003 [8,9]. According to these studies, obese AT is characterized by 40–60% macrophage accumulation compared with lean AT in which macrophages comprised only 10–15%. The release of various chemokines in obesity subsequently recruits elevated number of macrophages into AT and propagates a chronic inflammatory condition. Cytokines such as TNFα, IL-6, and IL-1β and many others stimulate paracrine pathways and accelerate inflammatory mechanisms. The increased number of macrophages infiltrating into AT constitutes a highly dynamic ATM population that is also a formidable source of inflammation [46].

### Heterogeneity of ATM in obesity

The population of ATM displays a wide-ranging diversity of phenotypes in continuum. The subtypes have been ascribed different nomenclature based on their distinctive signatures. The classical nomenclature involved M2/M1. The predominant profile in normal physiological condition is alternatively activated or M2 subtype secreting mostly anti-inflammatory cytokines viz. IL-4, IL-10, IL-13, etc. This is characterized by expression of CD206, CD301, CD68, and CD11b, Arginase1 and the common marker of macrophage, F4/80 [47]. M2-like ATM maintains tissue homeostasis by eradicating dead adipocytes [48]. Induction of adipocyte apoptosis in lean condition ushered in large M2 flux that was devoid of proinflammatory response. These macrophages possess capability to buffer lipid, which enables them to store lipids that are extruded from dead adipocytes and thereby prevent ectopic lipid accumulation and the ensuing lipotoxicity [49]. By inducing expression of UCP1, PGC1α and other browning genes, M2 polarization regulates thermogenesis and browning of WAT [50]. Moreover, alternatively activated M2 ATM is involved in adipogenesis [51]. M2 macrophages display up-regulation of genes involved in fatty acid oxidation and mitochondrial biogenesis [52]. These macrophages are characterized by production of the anti-inflammatory cytokine, IL-10, known for its role in insulin sensitivity. In a human study, the plasma concentrations of IL-10 showed strong positive correlation with insulin sensitivity [53]. The protective function of M2 macrophages in insulin resistance can be adjudged from the findings that higher levels of inflammation, weight gain, and insulin insensitivity are noticeable in mice with impaired M2 polarization [54,55].

Macrophage phenotypic switching is very prominent during obese condition; it is typified by the abundance of newly recruited and inflammatory macrophages referred to as classically activated M1 phenotype bearing F4/80, CD11c, and iNOS markers; M1 is elicited by interferon-γ (IFNγ) and typically releases proinflammatory factors, such as TNFα, IL-6, IL-1β, nitric oxide (NO), etc. [56]. The transition of M2 to M1 subtype causes chronic AT inflammation and insulin resistance [57]. M1 ATM mainly accumulates around dead adipocytes-forming CLS and exacerbates further infiltration of macrophages into inflamed AT unleashing a positive feedback loop that generates a chronic inflammatory ambience [56]. The copious secretion of cytokines can activate serine/threonine kinases like JNK (c-Jun N-terminal kinase) and IKK (inhibitor of NF-κB kinase) [58,59] that not only phosphorylate IR and IRS-1 interfering with normal insulin action but also instigate many transcription factors including c-Jun/Fos and NF-κB, which upon activation translocate to the nucleus and initiate transcription of inflammatory molecules [60].

Macrophages display varied preferences to use pathways to meet energy demands depending upon their phenotypes and functional needs [61]. M1 macrophages prefer aerobic glycolysis as the principal source for energy production, yielding two molecules of pyruvate and NADH from NAD^+^ along with ATP; conversion of pyruvate to lactate generates NAD^+^ to maintain glycolytic flux [62]. In M1-like subtypes, TCA cycle was seen to be inhibited at different steps owing to down-regulation of specific enzymes viz. isocitrate dehydrogenase and succinate dehydrogenase, resulting in greater accumulation of TCA cycle intermediates, citrate and succinate, respectively [63]. The accumulated succinate promoted stabilization of HIF-1α resulting in enhanced expression of proinflammatory and glycolytic genes [64,65]. Again, accumulated mitochondrial citrate can be exported to the cytosol through mitochondrial citrate carrier and cleaved into acetyl-CoA and oxaloacetate by ATP-citrate lyase (ACLY). From OAA, malate and ultimately pyruvate can be generated along with NADPH that produces increased NO and ROS in M1-like subpopulation that was confirmed by inflammatory cues elevating the expression of mitochondrial citrate carrier and ACLY [66,67]. Not only citrate but augmented pentose phosphate pathway (PPP) in M1-like phenotypes can be a source of NADPH that serves as a cofactor for iNOS to generate NO [68]. It was observed that NO hampered mitochondrial respiration (OXPHOS) and blocked M1 to M2 conversion [69]. Increased fatty acid uptake and triglyceride biosynthesis, and decreased lipolysis were also observed in M1 macrophages [70]. Conversely in M2-like phenotypes, the principal source of energy is OXPHOS-linked TCA cycle and fatty acid β-oxidation and decreased glycolysis, PPP and lipid biosynthesis [63]. But this conventional view is deemed not absolute as fatty acid oxidation is required to activate inflammosome complex in M1 subtypes, and fatty acid oxidation in M2 phenotypes is also dependent on glycolytic pathway [71].

ATM can also be classified into AT-resident macrophages and recruited monocyte-derived macrophages [72]. Previously, it was thought that bone marrow-derived circulatory monocytes are the main source of tissue-resident macrophages. Now, it is believed that in several tissues, the precursors of tissue-resident macrophages have already moved into those tissues during embryonic hematopoiesis before birth. These are self-renewing cells and circulatory monocytes are not required for their proliferation [73]. Sympathetic neuron-associated macrophages (SAMs), another type of macrophages, promote norepinephrine clearance upon uptake through Slc6a2 receptor and subsequently degrade it by the enzymatic action of monoamine oxidase A. Activation of sympathetic nervous system favors SAM’s proinflammatory phenotype through overaccumulation of norepinephrine. Deletion of Slc6a2 receptor not only prevents norepinephrine uptake but also obstructs development of obesity by inducing energy drainage through thermogenesis and fat browning [74]. Norepinephrine in normal condition promotes UCP1-dependent heat generation in mitochondria via uncoupling respiration from ATP generation [75]. Absence of Slc6a2 receptor helps to maintain the abundance of norepinephrine, thus promoting heat generation and reversal of obesity along with improving insulin sensitivity [37,74]. Trem2+ ATM subpopulation called lipid-associated macrophages (LAMs) have been identified in obese AT with functions in preventing adipocyte hypertrophy, inflammation, and metabolic dysfunction. The ablation of *Trem2* encourages metabolic disturbance only in high-fat diet-fed condition and not in control one [76].

Monocyte-chemoattractant protein-1 (MCP-1), released from hypertrophic obese adipocytes, helps the monocytes to permeate AT and promotes further macrophage differentiation. Bone marrow-derived M0 macrophages either give rise to proinflammatory M1-like subpopulation or anti-inflammatory M2 subtypes depending on which stimuli they receive. Several cytokines, adipokines, different signaling molecules like IFNγ, IKK, JNK, and specific protein receptors such as peroxisome proliferator-activated receptors (PPARγ, PPARδ) play major role in phenotypic switching [77]. Based on the stimuli, M2 macrophages are further differentiated into M2a, M2b, M2c; the interleukins IL-4, IL-13 stimulate M2a, TLRs are the stimulators of M2b while M2c is stimulated by IL-10 and transforming growth factor-β (TGF-β), all of which have different anti-inflammatory functions in AT [78].

**Figure 2:**
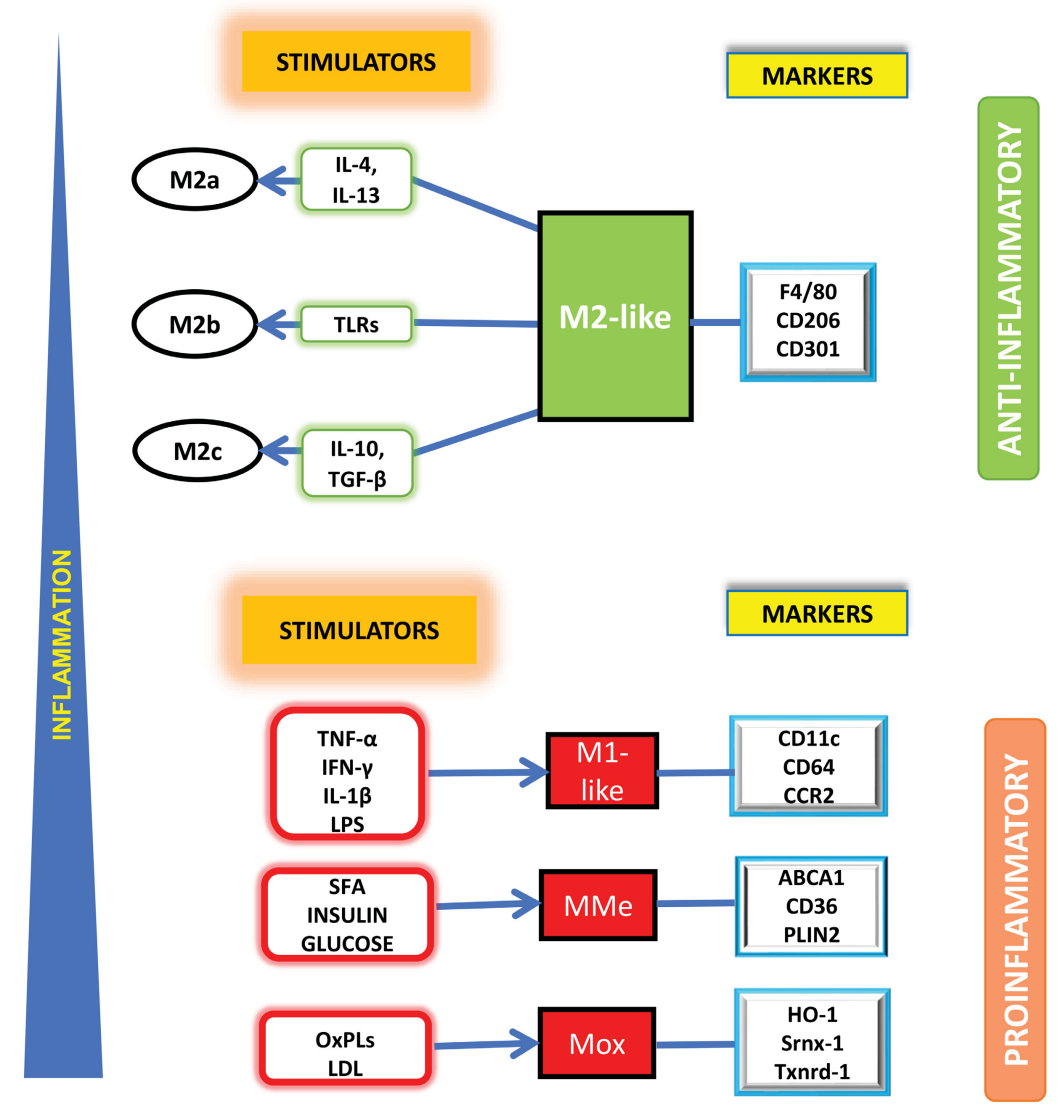
Changes in ATM composition with increasing inflammation During conditions of lesser inflammation, the anti-inflammatory M2-like ATM phenotype with F4/80, CD206, CD301 markers, becomes abundant and gives rise to three basic subtypes M2a, M2b, M2c depending on the available stimuli viz. IL-4 and IL-13 for M2a; TLRs for M2b and IL-10 and TGF-β for M2c. During conditions of heightened inflammation, the proinflammatory M1-like ATM becomes predominant and different subtypes viz. MMe and Mox subpopulations of ATM with different specific markers arise stimulated by varied cues emanating from the proinflammatory AT microenvironment.

In addition, two novel macrophage phenotypes have been reported to be involved in the development of obesity-induced insulin resistance, namely, metabolically activated (MMe) macrophages and oxidized macrophages (Mox) [77]. MMe is induced by SFA or high levels of insulin [79] having both pro- and anti-inflammatory features. It regulates obesity-induced AT inflammation and clears dead adipocyte debris [77]. Oxidized phospholipids (OxPLs) induce development of another macrophage subtype, Mox; oxidative tissue damage mediates overexpression of Nrf2, which plays crucial role in the development of Mox phenotype [80]. The different ATM subpopulations bearing distinctive markers respond to varied stimuli and have characteristic proinflammatory or anti-inflammatory attributes as shown in Figure 2 adapted from [78].

Previously, higher and lower abundance of CD11c marker was the basis of macrophage subdivision. But this concept is not absolute as the expression level of this marker varies among ATM subpopulations. A recent report propounded the concept of classifying ATM in a new way and subdivided ATM into three subgroups: CD9+, CD9^‒^, and Ly6c^+^; the Ly6c ATM is adipogenic in nature localized mostly on outer sides of CLS, whereas proinflammatory CD9 ATM are lodged inside CLS [81]. MFe^hi^, another type of alternatively activated ATM, is rich in iron having up-regulated expression of iron-recycling genes while rest of the ATM are denoted as MFe^lo^. Normally near about 25% of all ATM are MFe^hi^ in lean subjects, whereas its level declines in obese models due to enhanced prevalence of MFe^lo^ [82]. Most commonly in human AT, proinflammatory ATM is characterized as CD14^+^ CD16^+^ CD36^high^, while anti-inflammatory subtypes are CD14^+^ CD16^‒^ CD163^+^ [83]. Recent experiments have shown that on the basis of MHCII and CD11c expression, ATM can be grouped into F4/80^hi^MHCII^+^CD11c^‒^ and F4/80^hi^MHCII^+^CD11c^+^ subpopulations that exhibit higher turnover rate in obese condition [84]; the researchers have identified fetal-derived ATM as Tim4^+^CCR2^‒^MHCII^+^CD11c^‒^, while monocyte-derived ATM are Tim4^‒^CCR2^+^ MHCII^+^CD11c^‒^, and the latter replace the former in HFD-fed obese mice.

### Triggers for macrophage infiltration into inflamed AT and their polarization

In conditions of caloric excess, the storage of energy in AT is facilitated by both adipocyte hypertrophy and hyperplasia [85]. Adipocyte expansion ultimately leads to hypoxia, cell death, fat leakage, and altered secretion of adipokines, all of which create favorable environment for macrophage accumulation and could be potential triggers for macrophage infiltration [86].

#### Hypoxia

Angiogenesis is required to maintain proper oxygen supply in gradually expanding AT with energy overload. When the rate of hyperplasia is fast, then the developing vasculature fails to compensate deficiency of oxygen [87] and may lead to adipocyte death and fat leakage [85]. Cellular responses to low oxygen are majorly regulated by the transcription factor, hypoxia-inducible factor alpha (HIF-1α); continuous synthesis and degradation of HIF-1α takes place in normoxic condition, but during hypoxia degradation of HIF-1α is prevented that allows its nuclear translocation and further activation of downstream genes not only of angiogenesis but also inflammation and energy metabolism [88,89]. It was noticed that deletion of adipocyte-specific *Hif-1α* prevented obesity-linked inflammation and insulin resistance [90]. The region of adipocyte necrosis becomes generally hypoxic. Various endogenous DAMPs attract ATM for clearance of necrotic adipocytes. Hypoxia plays a crucial role in ATM polarization to inflammatory subtypes [91]. According to these researchers, HFD-fed mice displayed higher signal of pimonidazole (indicative of hypoxia) and increased HIF-1α expression in epididymal adipose depot than wild type. A population-based study on human subjects has revealed lower partial pressure of oxygen in AT in obese subjects compared with the lean, concomitant with inflammatory responses in obese condition [92]. Prolonged unresolved hypoxia can lead to fibrosis, cellular senescence, and necrotic adipocyte death [93].

#### Fibrosis

During the course of obesity, interstitial fibrosis in AT occurs from abnormal accumulation of extracellular matrix (ECM) components, which can potentially trigger AT dysfunction through inflammation and macrophage infiltration [86,94]. Up-regulated HIF-1α expression in AT enables augmented synthesis of ECM components initiating fibrosis. In addition, HIF-1α can also influence collagen cross-linking and stability by regulating the key enzymes viz. lysyl oxidase (LOX) and prolyl-4-hydroxylase [95]. ATM are critically involved in fibrotic process through mediators such as transforming growth factor-β1 (TGF-β1) and platelet-derived growth factor (PDGF) that can directly activate fibroblasts and regulate ECM [96]. ATM-derived factors can attract fibroblasts and other inflammatory cells. A causal relationship between ECM and inflammation in obesity was highlighted by evidences of augmented collagen VI α3 subunit mRNA along with enhanced macrophage chemotaxis and M1 ATM prevalence in obese human subjects, which was eventually lowered upon treatment with the thiazolidinedione (TZD)-type antidiabetic drug, pioglitazone [97]. Furthermore, collagen VI-null mice exhibited marked reduction in macrophage infiltration in AT [98]. AT fibrosis is complex but closely linked to insulin resistance [99]. The role of ATM in this is definitive as M1 macrophages being primary producers of TNFα, iNOS, and IL-6, promote proinflammatory and profibrotic phenotype of adipose-derived stem cells accentuating AT inflammation and fibrosis [100,101].

#### Adipocyte death

Necrosis is prevalent to a considerable extent in lipid-laden adipocytes and provides a strong stimulus to macrophages to get recruited to the necrotic sites. Macrophages come to engulf the lipid spilled from adipocytes after necrosis, and form CLS around dead adipocytes [102]. Both apoptosis and necrosis of adipocytes can promote CLS formation [86,103]. In fact, the mode of cell death is insignificant factor here as adipocyte death alone was shown to promote CLS formation even in lean AT without any pre-existing inflammation. When AT from lean persons received laser injury in *ex-vivo* culture, it acted as a perfect model for apoptosis and ATM was observed to form CLS around dead tissue after 10 h, which reached its peak at 60 h [103].

#### Fat leakage

After reaching a certain level of expansion, fat leaks out from adipocytes [104] and becomes surrounded by ATM. The size of lipid droplets decides the behavior of ATM around it. Small-sized (25 μm) droplet is efferocytized by single ATM, whereas medium-sized (25–50 μm) is efferocytized by ATM after processing; the size of droplet is 100–200 μm in case of obese, and not classically efferocytized but phagocytosed by the formation of CLS [103]. Efficient efferocytosis by anti-inflammatory macrophages clears the dead adipocytes and is essential for proper cellular turnover and tissue homeostasis [105]. However, in lipid-laden AT, macrophages forming CLS have distinctive proinflammatory attributes [106]; the ensuing inefficient adipocyte clearance is a factor contributing to AT inflammation.

#### Adipokines/chemokines

Adipocytes are known to secrete an array of cytokines/chemokines collectively called adipokines, some of which can act as chemotactic signals for macrophages. One such candidate molecule is monocyte chemoattractant protein-1 (MCP-1)/chemokine (C–C motif) ligand (CCL2), a member of the C–C chemokine family [107]. Obese subjects have higher levels of adipocyte MCP-1 that is reduced following weight loss [108]. Human obese subjects registered higher circulating levels of MCP-1 that in turn, positively correlated with several obesity-related parameters [109]. Augmented MCP-1 expression could drive macrophage infiltration into obese AT [110]. On a similar note, increased ATM content was noticed in transgenic mice overexpressing MCP-1 and it could contribute toward insulin resistance [111]. Chemotaxis of bone marrow macrophages (BMC) in obese AT was found to be MCP-1-dependent and mediated through binding to its receptor CCR2 on BMC [112]. In spite of HFD feeding, CCR2^−/−^ mice displayed lesser infiltrated macrophages than CCR2^+/+^. Migration of BMC was higher in *ob/ob*-adipocyte conditioned media than that of wild type. At the same time, MCP-1 concentration was higher in the former than the latter [112]. Targeting CCR2-associated GPI-anchored protein with propagermanium in human monocytic cell line inhibited MCP-1-induced macrophage migration [113]. In addition, MCP-1 deficiency enhanced M2 macrophages and revived insulin sensitivity [114]. However, fluorescently-labeled monocyte tracking revealed MCP-1 as only partially potent chemoattractant [115]. According to this report, ATM content was reduced by approximately 40% when labeled monocytes from CCR2 KO mice were injected into wild-type recipients, or labeled wild-type monocytes were injected into MCP-1 KO mice. On the other hand, MCP-1 action was more effective in the liver as deletion of CCR2/MCP-1 system led to about 85% decrease in recruited hepatic macrophages [115]. This important experimental evidence pointed toward presence of other chemotactic signal(s) besides MCP1. Another adipokine, CXCL12 (stromal cell-derived factor 1) that is extensively secreted from hypertrophied adipocytes recruited macrophages into AT [116]. At higher levels in obese models, the hepato-adipokine FetA proved an effective signal for macrophage chemotaxis and acted synergistically with MCP-1 [27]. Furthermore, in a transculture setup of adipocytes and macrophages, addition of the SFA palmitate to adipocytes released FetA into the medium, which could drive polarity shift in macrophages from anti-inflammatory phenotype to proinflammatory subtype [27]. A key regulator of macrophage activation is IFNγ; a distinct M2-shift in ATM phenotype and cytokine expression accompanied by decrease in adipocyte size and improved insulin sensitivity were observed in obese IFNγ-KO mice [117].

#### Intercellular mitochondria transfer

Recent studies have highlighted intercellular mitochondria transfer from the adipocytes to macrophages within AT as a homeostatic mechanism regulating local AT microenvironment [118,119]. The internalization of mitochondria by ATM from adipocytes is facilitated by heparan sulfate; deletion of genes-encoding enzymes involved in heparan sulfate biosynthesis was found to impair mitochondria uptake by ATM, and caused glucose intolerance and lower energy expenditure [118]. These authors also demonstrated how macrophages with acquired mitochondria were transcriptionally quite distinct from macrophages, which did not take up mitochondria, thus forming a distinct ATM subpopulation. Furthermore, in obese murine models, the proinflammatory M1-like polarization was associated with reduced mitochondria uptake, while M2-like ATM exhibited higher rate of intercellular mitochondria transfer [118]. The dietary long-chain fatty acids have been observed to inhibit mitochondria uptake by ATM [120].

## Transcriptional regulation of ATM: the key mechanisms

### NF-κB

NF-κB is a family of five related proteins, named RelA (p65), RelB, c-Rel, NF-κB1 (p105/50), and NF-κB2 (p100/52). All these proteins contain a conserved sequence named Rel homology domain (RHD) at their N-terminal regions responsible for dimerization and binding to target DNA. Homodimers of p50 and p52 act as transcriptional repressors as they lack transactivation domain (TAD) in their C-terminal region for inducing target gene expression, which is typically present in other three (p65, RelB, c-Rel) proteins [121]. In the cytosol, association with IκB protein renders NF-κB inert. Upon receiving stimulus, phosphorylation and degradation of IκB unmasks the nuclear localization signal (NLS) of NF-κB paving its nuclear translocation for gene activation [122]. Such stimuli-induced phosphorylation is mediated via IκB kinase (IKK) comprising IKK-α, IKK-β, and other scaffold proteins [123].

NF-κB is a key transcriptional regulator of M1 macrophage polarization induced by TLR4. This is asserted by TLR4-deficient mice fed high-fat diet showing markedly resolved AT inflammation and predominant M2-like subtypes [124]. Activation of TLR4 signaling acts through IKK-mediated phosphorylation of IκB. Adaptor protein myeloid differentiation primary response gene 88 (MyD88) is an important player in proinflammatory cytokine expression, while MyD88-independent pathway drives type I interferons (IFN) and IFN-inducible gene expression. Both pathways eventually activate IKK leading to phosphorylation and degradation of IκB, thereby promoting nuclear localization of NF-κB [125].

NO has been shown to promote the activity of NF-κB and thus regulates TLR4-mediated inflammatory response [126]. Suppressor of cytokine signaling (SOCS1) acts as ubiquitin ligase and stifles NF-κB-mediated inflammatory response by degrading DNA-bound p65 subunit of NF-κB [127]. NO derived from nitric oxide synthase 1 (NOS1) stabilizes NF-κB by causing S-nitrosation of SOCS1 by which it loses its p65-binding capacity and gets degraded. Thus, NO facilitates NF-κB-mediated control of proinflammatory cytokines at transcriptional level [126].

### AP-1

Activated protein-1 (AP-1) exists as heterodimeric complex of two proteins, which are products of two protooncogenes, *c-jun* and *c-fos*. This heterodimer with the help of specific DNA-binding capacity of c-Jun binds to the promoter containing AP-1 activation elements to elicit the effect of target genes [128]. TLR4 promotes M1-like phenotypes of macrophages not only by activating the transcription factor NF-κB but also AP1. The TLR4-NOS1-AP1 axis has a role in this polarization event. Activated TLR4 induces NO production from NOS1 and causes conformational change in ATF2 via cysteine nitrosylation, stabilizes the Fos-Jun dimer of AP1 rather than Jun-ATF2 dimer, although the inhibition of NOS1 stabilizes the alternative form, Jun-ATF2 dimer. Fos-Jun dimer is better at regulating the inflammatory response than Jun-ATF2 dimer as the former binds strongly to the regulatory region of inflammatory genes (IL-12, IL-23) than the latter [129].

### STATs

ATM polarization toward M1-like phenotypes is also mediated via JAK-STAT pathway. The number of mammalian STAT is 7 (STATs 1–4, 5A, 5B, and 6) and JAK is 4 (JAKs 1–3 and Tyk2) with significant tissue-specific differences in abundance and function [130]. STATs are associated not only with M1 activation but also with M2 stimulation; STAT1 and 5 induce M1-like polarization, while STAT3 and 6 promote anti-inflammatory M2-like subtypes [131]. Binding of ligand to the membrane-bound receptors activates receptor-linked JAKs leading to phosphorylation of Tyr residues in the receptors. STAT is recruited to the system and attaches to the activated receptor through its –SH2 domain. Then, JAKs catalyze phosphorylation of any one of the C-ter Tyr residue STATs, following homo-/heterodimerization, which facilitates nuclear translocation [130]. IFNγ could induce M1-like macrophage polarization via JAK-STAT pathway [132]. In the context of AT inflammation, we found that FetA played a vital role by polarizing ATM toward M1-like subtypes through heightened expression of MCP-1 and iNOS via JNK-cJun-IFNγ-JAK2-STAT1 signaling cascade [133].

### FoxO1

PAR2 is another receptor known to cause ATM polarization [134] and mediates its effect through the activation of transcription factor FoxO1. PAR2-induced up-regulated expression of proinflammatory cytokines in macrophages get diminished in FoxO1-silenced condition [135]. PAR2 activation involves irreversible proteolytic mechanism; PAR2 is a unique GPCR, unlike the traditional one, protease-mediated cleavage of its extracellular N-ter exposes a new site that binds intramolecularly for receptor activation and signal transduction [136]. The phosphorylated form of FoxO1 is inert in cytoplasm and maintained by PI3K-AKT signaling [137]. Its nuclear localization is important to conduct the downstream effects, which are mediated by pSTAT3 [115]. In obese condition, expression of PAR2 is up-regulated in adipocytes and macrophages. Treatment with its antagonist exerts reverse effect on adiposity and inflammation [134]. PAR2 agonist promotes STAT3 phosphorylation and PAR2-dependent FoxO1-mediated ATM polarization to M1-like phenotype is conducted through JAK2/STAT3 pathway [135].

### IRFs

Another vital transcription factor for macrophage polarization is the family of interferon regulatory factors (IRFs) comprising nine members with nearly conserved N-ter DNA-binding domains. Except IRF6, all other members contain C-ter IAD domain that enables their association with each other. Of these, IRF1–5 and IRF8 promote macrophage differentiation and polarization [138,139]. IRF5 is a key mediator for generating immune responses through proinflammatory cytokines and acts downstream of TLR-MYD88-signaling pathway [140]. TLRs respond to a wide variety of ligands termed PAMPs or DAMPs; the interaction occurs with TLR homo- or heterodimers along with a coreceptor or accessory molecule [141]. Ligand binding to TLR7 and TLR9 activates the transcription factor IRF5. TRAF6-mediated K63-linked ubiquitination at 410^th^ and 411^th^ lysine residues of IRF5 is important for its activation and proper nuclear translocation. For this ubiquitination-mediated activation, at first association of IRF5 with IRAK1 is a prerequisite [142]. Phosphoryaltion at Ser 451 and 462 is required for its dimerization and accurate transportation to nucleus [143]. Although IRF5 encourages M1-like subtype progression, in the presence of IRF4, it competes with IRF4 to interact with MYD88, a downstream molecule of TLR-mediated signaling pathway, and promotes M2-like phenotype [144]. The inhibitory domain of C-ter of IRF4 promotes autoinhibition by interacting with its N-ter DNA-binding domain. Interaction between pSer-148 of the PEST region of PU.1 with K399 of IRF4 promotes unmasking of IRF4’s DBD domain and the subsequent interaction between DBD domain of PU.1 and IRF4 is also required [145,146]. M2 marker genes are repressed by Polycomb group-mediated histone methylation at positions H3K4 and H3K27 on their promoter region. IL-4 stimulus promotes STAT6 activation and nuclear localization to activate *Jmjd3* gene expression; *Jmjd3* causes demethylation of H3K27 and relieves repressed M2 marker gene expression [147,148]. Jmjd3–IRF4 axis is considered important for M2 polarization [149].

Phosphorylations at two sites (between 138–150 amino acids and C-ter 219–231 amino acids) by casein kinase II make IRF1 active [150]. Inhibitory phosphorylation (215/219/221 positons) by IκB kinase-ε prevents the interaction of IRF1 with NF-κB to its RelA subunit by structural modification, thus inhibiting its transcriptional activity [151]. IRF1 also acts as a downstream molecule of IFNγ-induced TLR4 pathway by interacting with MyD88 [152] up-regulating proinflammatory gene expression [153]. IRF1 promotes M1-like phenotypes, but with the help of IRF2 it represses proinflammatory gene expression [154]. IRF1 and 2 both can recognize IFNγ-induced genes and IRF2 competes with and represses IRF1-mediated gene expression [155]. Actually, in this context, the role of IRF2 is somehow complex and contradictory. Up-regulation of IFNγ, IL-6, IL-1, IL-12 was induced by IRF2 while at the same time it inhibited the expression of TNFα [156]. Phosphorylation at site 2 of IRF3 by IKKε or TBK1 relieved the autoinhibition and allowed its interaction with CBP/p300, which ultimately promoted the phosphorylation at site 1. This is important for their dimerization and translocation into the nucleus to boost target gene expression [157]. IRF3 facilitates M2-like phenotypes by stimulating PI3K-AKT pathway [158].

### PPARs

Peroxisome proliferator-activated receptors (PPARs) are ubiquitous ligand-activated transcription factors that are expressed in various metabolic tissues. These are critically involved in many metabolic processes including terminal differentiation of some cell types. In recent years, their regulation has been reported in macrophage-mediated immune response. There are three mammalian subtypes of PPARs, i.e., α, β/δ, and γ.

#### PPARα

Anti-inflammatory function of PPARα makes it a potent inhibitor of macrophage inflammation in obese condition. It strongly suppresses other inflammatory signaling pathways like AP-1 and NF-κB to inhibit macrophage-mediated inflammation [159]. Secretion of MMP-9, a major proinflammatory molecule, is inhibited when PPARα is activated by using its agonist [159]. A report demonstrated that Z-551, a PPARα agonist ameliorates high-fat diet-induced obesity and metabolic disorders in mice, and one of the mechanisms by which it exerts its effect is by reducing ATM that ultimately suppresses AT inflammation [160]. While common macrophage markers (Emr1, Cd68), M1 macrophage markers (Ccl2, Tlr4) were significantly reduced by PPARα agonist, it had no effect on the M2 macrophages [160]. PPARα activation could lower fat accumulation inside macrophages, a major cause for macrophage inflammation and this is directly linked to another important metabolic disorder, atherosclerosis [161].

#### PPARβ/δ

PPARδ plays important role in macrophage class switching. The secretion of anti-inflammatory cytokines from adipocytes such as IL-4 and IL-13 tips the phenotypic polarization toward M2; but IL-4 and IL-13 cannot mediate their anti-inflammatory effect upon the PPARδ-ablated ATM indicating PPARδ is necessary for phenotypic transition of macrophages toward M2 [54]. PPARδ has been reported to suppress inflammation in macrophages that is also associated with the stimulation of fatty acid catabolism [162,163]. PPARδ-influenced macrophage polarization toward M2 is not only restricted to AT but also has been seen in the bone marrow, indicating the role of PPARδ in the regulation of overall inflammation [164].

#### PPARγ

Two isoforms of PPARγ, γ1 and γ2, are highly expressed in AT and become suppressed in diet-induced obesity [165,166]. Activation of PPARγ is associated with reduced AT inflammation [167]. PPARγ aids the release of adiponectin both from adipocytes and macrophage and this is one of the mechanisms by which it exerts its anti-inflammatory effects on AT [168,169]. IFNγ acts as a potent regulator of macrophage polarization toward M1 phenotype inside inflamed AT [133]. The antidiabetic PPARγ ligand TZD strongly suppresses the interferon expression reducing the M1 macrophage number [8]. Besides, TZD significantly reduces proinflammatory cytokine expression (IL-6, TNFα, IL-1) from AT-resident macrophages [8,9]. PPARγ ligand suppresses MCP-1, the potent chemoattractant for ATM that results in the reduction in total macrophage content in AT. Pioglitazone, another PPARγ ligand, also reduces ATM number improving insulin sensitivity in obese subjects [170]. The influence of preadipocytes in this context was highlighted by a study showing that NF-κB and MAPK-mediated heightened expression of proinflammatory cytokines/chemokines in preadipocytes cause suppression of PPARγ activity in human adipocytes [171].

## Concluding remarks

During the development of obesity, the AT microenvironment undergoes drastic transformations skewing it toward a proinflammatory status. ATM is a highly heterogeneous cell population varying not only in phenotypic characteristics but also in frequency and origin [84]. High-fat diet not only augments ATM content but also drives increased recruitment of unique macrophage subpopulations that are one of the major mediators of inflammation within lipid-laden AT paving the onset of metabolic syndrome. However, apart from potentiating inflammation, ATM exerts crucial beneficial effects during obesity including induction of lysosomal genes in ATM [172], stimulation of lysosomal exocytosis along with enhanced expression of lipid metabolism genes to handle the excess fat in obese AT, while inflammatory mediators facilitate recruitment of additional macrophages to CLS enabling efficient clearance of dying adipocytes [173]. A recent elegant series of experiments performed using targeted *in vivo* ATM ablation revealed diverse functions of AT-resident macrophages in maintaining vascular integrity and controlling adipocyte hypertrophy, secretion, and fibrosis in AT thereby restraining AT dysfunction that is a typical pathophysiologic feature of obesity-associated insulin resistance [174]. The role of ATM as friend or a foe is determined by a complex interplay of factors arising within lipid-enriched AT niche.

## Future perspectives

Several studies have extensively highlighted the involvement of ATM in obesity-induced insulin resistance. A brief summary of the key research findings in the past two decades that have immensely contributed in our understanding of ATM biology in the context of obesity-associated insulin resistance is presented in Table 1. Nevertheless, there are still several unexplored domains. As obesity is strongly associated with various metabolic diseases, deciphering the impact of obesogenic stimuli in regulating ATM dynamics would provide crucial insights into guiding macrophage-based therapeutic approaches. Besides, migration and accumulation of macrophage in obese subjects is not specific to AT but occurs in other organs as well. The potential interorgan cross-talk is now evident and requires further in-depth analyses. Considering the active pace of research in this area, the next few years would uncover still important aspects of macrophage function, metabolism, and activation during the progression of obesity. At the current level of understanding, we can only marvel and appreciate the complex dynamics of ATM in the scenario of obesity-mediated insulin resistance.

**Table 1:** Highlights of key research advances in understanding ATM biology in obesity-associated insulin resistance over the past two decades

Key research advances	Sources
● Number of cells positive for macrophage marker F4/80 is directly proportional to the size and mass of AT depot. ● The expression of a host of cytokines viz. TNFα, iNOS, IL-6 gets exacerbated in obese AT due to recruited ATM generating a proinflammatory milieu.	Weisberg et al., 2003 [8]
● In obese condition, high-circulatory insulin concentration, ATM invasion, and subsequent inflammation in white AT go hand in hand, which establish insulin resistance as an inflammatory disease.	Xu et al., 2003 [9]
● Oversupply of MCP-1 from obese AT modulates its adverse effects in paracrine and endocrine manner. ● Transgenic mice model with MCP-1 overexpression (aP2-MCP-1) not only blunts the insulin-signaling pathway but also inhibits glucose clearance from blood and promotes glucose production within the cell.	Kamei et al., 2006 [111]
● The overexpression of MCP-1 in obese mice also causes hepatic steatosis along with ATM recruitment and insulin resistance.	Kanda et al., 2006 [110]
● In obese AT, CD11c^+^ myeloid cells become highly abundant to instigate inflammatory responses. Treatment with free fatty acids can activate those cells, triggering inflammation, and further worsening insulin resistance through TLR2/4-JNK-signaling cascade.	Nguyen et al., 2007 [60]
● Not only *Ccl2 /Mcp-1* but a host of molecules act in concert on the obese AT to promote ATM infiltration and subsequent inflammation. Naturally, the ablation of *Ccl2 /Mcp-1* alone cannot show any reversal effect. ● Moreover, CCL2 can influence various metabolic events that seem not dependent on ATM recruitment.	Inouye et al., 2007 [175]
● In lean AT, a novel population of ATM marked F4/80^+^CD11c^+^ is absent, although present in obese mice. In lean state, alternatively activated M2-like phenotype is predominant with accelerated expression of *arginase1, IL-10* genes while the M1-like classically activated subpopulation of macrophages with extensive expression of *TNF-α, iNOS* is prevalent in obese AT. ● The phenotypic shifting toward M2-like subtype was favored in *Ccr2*-KO obese mice, which reveal the importance of this receptor in this context.	Lumeng et al., 2007 [176]
● In obese mice, ATM becomes lipid-laden with enhanced expression of lipid-handling genes (*Pparγ, Adfp, Apob48r*).	Lumeng et al., 2007 [177]
● Infiltrated macrophages help in the leaching of SFAs from hypertrophied adipocytes. These free fatty acids through TLR4-NF-κB signaling pathway generate inflammatory ambience in adipocytes and macrophages.	Suganami et al., 2007 [178]
● Spatial and temporal variations are the basis for the infiltration of different ATM subpopulations, which also generate the M2a/M1 equilibrium. In lean subjects, M2a resides in interstitial region of AT but with developing obesity, infiltration of M1-like subtypes into necrotic adipocytes gets augmented; M1 are mainly appointed from circulation rather than conversion from M2a subtype of interstitial space.	Lumeng et al., 2008 [106]
● Knockout of *Ikkε* gene promotes improvement of whole-body metabolic status by decreasing the inflammatory cytokines expression and increasing the energy drainage. This also alters the expression of glucose- and lipid-metabolizing enzymes. ● As the absence of IKKε resolves the obesity-linked metabolic complications associated with insulin resistance and T2D, it can be used as therapeutic target for recovery in future.	Chiang et al., 2009 [179]
● Reduction of adipose Treg (regulatory T cells) occurs in mice and humans during obesity. The depletion of local differentiation plays major role in this reduction than impaired homing. ● Tregs can act as the indicators in adipose inflammation.	Deiuliis et al., 2011 [180]
● Foam cells produced during atherosclerotic lesion are often confused with fat-laden ATM of obese subjects. Although the mechanism of macrophage infiltration and subsequent inflammation are not same in those two cases. ● CX_3_CR1 fosters foam cell formation in high-fat diet-induced obese mice; CX_3_CR1 is not needed for the ATM infiltration.	Morris et al., 2012 [181]
● TLR4-mediated signaling in hematopoetic cell is important for M1-like phenotype development; its deletion favors the progression toward M2-like subtype. This improves the inflammatory status of the body but not the insulin sensitivity.	Orr et al., 2012 [124]
● CCR2/MCP-1 system is crucially involved in the migration of monocytes into AT and also regulating the appearance of infiltrated macrophages in the liver. ● Ablation of either CCR2 or MCP-1 discourages the accumulation of macrophages both in AT (∼40%) and liver (∼80%) with varying extent. ● CCR2 or MCP-1 has greater impact on liver macrophage infiltration rather than ATM recruitment, which reveals that latter is controlled by other factor(s) besides CCR2/MCP-1 system.	Oh et al., 2012 [115]
● During obesity, hypertrophied adipocytes induce biogenesis of lysosomes in ATM independently from inflammatory responses. It helps in lipid catabolism and lipid trafficking. Absence of this program disturbs the lipid metabolism in ATM and causes the accumulation of lipids in it.	Xu et al., 2013 [172]
● In hyperlipidemic condition, fatty acid-TLR4-NF-κB signaling pathway up-regulates the expression of FetA, an adipokine. FetA acts in a synergistic manner with MCP-1 to stimulate ATM infiltration and accelerates its polarization to M1 subtypes via TLR4-mediated pathway.	Chatterjee et al., 2013 [27]
● NK cell promotes macrophage recruitment in intra-abdominal adipose repository but not in subcutaneous adipose depots and spleen. NK deletion improves the insulin sensitivity without altering the levels of inflammatory cytokines.	O’Rourke et al., 2014 [182]
● Although weight loss is a well-accepted therapy for improving metabolic disturbance, it fails to fully mitigate the AT inflammation because the proliferation of ATM is sustained during the weight-loss process, which develops insulin resistance.	Zamarron et al., 2017 [183]
● Genetic ablation of Trem2^+^ LAM subpopulation adversely affects lipid homeostasis and leads to adipocyte hypertrophy, excess cholesterol and body fat, and insulin resistance.	Jaitin et al., 2019 [76]
● Lean and obese AT have characteristically different ATM subpopulations that differ in their developmental origins and turnover rates. ● Obesity causes AT remodeling which, in turn, can influence ATM distribution and frequency within AT. ● Mostly, fetal-derived Tim4^+^CCR2^‒^MHCII^+^CD11c^‒^ ATM are replaced by monocyte-derived Tim4^‒^CCR2^+^MHCII^+^CD11c^‒^ATM.	Chen and Reudl, 2020 [84]
● FetA plays a pivotal role in developing AT inflammation through ATM polarization by regulating the expression of MCP-1 and iNOS via JNK-cJun-IFNγ-JAK2-STAT1 signaling cascade.	Chattopadhyay et al., 2021 [133]
● In obese AT, CD11c+ ATM regulates lipid metabolism. The AT-resident macrophages support vascular integrity and restrain obesity-induced fibrosis, thereby playing a protective role in buffering white AT from obesity-driven pathological remodeling.	Chen et al., 2021 [174]

## Abbreviations

ABCA1: ATP-binding cassette transporter A1
ACLY: ATP-citrate lyase
*Adfp*: *adipose differentiation-related protein*
AHSG: alpha2 Heremans-Schmid glycoprotein
AP-1: activated protein-1
*Apob48r*: *apolipoprotein B48 receptor*
AT: adipose tissue
ATF2: activating transcription factor 2
ATM: adipose tissue macrophage
ATP: adenosine triphosphate
BAT: brown adipose tissue
BMC: bone marrow macrophage
CBP: CREB-binding protein
CCL2: C-C motif chemokine ligand 2
CCR2: C-C chemokine receptor type 2
CD: cluster of differentiation
CLR: C-type lectin receptor
CLS: crown-like structure
CREB: cAMP-response element binding
CX_3_CR1: C-X3-C motif chemokine receptor 1
CXCL12: C-X-C motif chemokine ligand 12
DAMP: damage-associated molecular pattern
DBD: DNA-binding domain
ECM: extracellular matrix
FA: fatty acid
FetA: fetuin-A
FFA: free fatty acid
FoxO1: forkhead box protein O1
GLUT4: glucose transporter type 4
GPCR: G-protein-coupled receptor
GPI: glycosylphosphatidylinositol
H3K27: histone 3 lysine 27
H3K4: histone 3 lysine 4
HFD: high fat diet
HIF-1α: hypoxia-inducible factor-1α
HMGA1: high mobility group A1
HO-1: heme oxygenase-1
IAD: IRF-association domain
IFNγ: interferon γ
IKK: inhibitor of NF-κB kinase
IL: interleukin
iNOS: inducible nitric oxide synthase
IR: insulin receptor
IRAK: interleukin-1 receptor-associated kinase 1
IRF: interferon regulatory factor
IRS-1: insulin receptor substrate-1
IκB: inhibitor of nuclear factor κB
JAK: Janus kinase
Jmjd3: Jumonji domain containing-3
JNK: c-Jun N-terminal kinase
KO: knockout
LAM: lipid-associated macrophage
LDL: low-density lipoprotein
LOX: lysyl oxidase
LPS: lipopolysaccharide
MAO A: monoamine oxidase A
MAPK: mitogen-activated protein kinase
MCP-1: monocyte chemoattractant protein-1
MMe: metabolically activated macrophage
MMP-9: matrix metalloproteinase-9
Mox: oxidized macrophage
MyD88: myeloid differentiation primary response 88
NAD: nicotinamide adenine dinucleotide
NADP: nicotinamide adenine dinucleotide phosphate
NE: norepinephrine
NF-κB: nuclear factor kappa B
NLR: NOD-like receptor
NLS: nuclear localization signal
NO: nitric oxide
NOD: nucleotide-binding oligomerization domain
NOS1: nitric oxide synthase 1
Nrf2: nuclear factor erythroid 2–related factor 2
OAA: oxaloacetic acid
OXPHOS: oxidative phosphorylation
OxPL: oxidized phospholipid
PAMP: pathogen-associated molecular pattern
PAR2: protease-activated receptor 2
PDGF: platelet-derived growth factor
PGC1α: peroxisome proliferator-activated receptor-gamma coactivator-1 alpha
PI3K: phosphatidylinositol 3-kinase
PKC: protein kinase C
PLIN2: perilipin 2
PPAR: peroxisome proliferator-activated receptor
pPKCε: phospo-protein kinase C epsilon
PPP: pentose phosphate pathway
RHD: Rel-homology domain
RIG-I: retinoic acid-inducible gene I
RLRs: RIG-I-like helicase receptors
ROS: reactive oxygen species
SAM: sympathetic neuron-associated macrophage
SFA: saturated fatty acid
SH2 domain: Src homology 2 domain
Slc6a2: solute carrier family 6 member 2
SNS: sympathetic nervous system
SOCS1: suppressor of cytokine signaling 1
Srnx-1: sulforedoxin-1
STAT: signal transducer and activator of transcription
T2D: type 2 diabetes
TAD: transactivation domain
TBK1: tank-binding kinase 1
TCA cycle: tricarboxylic acid cycle
TGF-β: transforming growth factor-β
TLRs: toll-like receptors
TNFα: tumor necrosis factor α
TRAF6: tumor necrosis factor receptor-associated factor 6
Treg: regulatory T cell
Txnrd-1: thioredoxin-1 reductase
Tyk2: tyrosine kinase 2
TZD: thiazolidinedione
UCP1: uncoupling protein 1
*Vegfa*: *vascular endothelial growth factor A*
WAT: white adipose tissue
